# The Prevalence of Sleep Apnea in Iran: a Systematic Review and Meta-Analysis

**Published:** 2019-01

**Authors:** Mandana Sarokhani, Mitra Goli, Shahin Salarvand, Reza Ghanei Gheshlagh

**Affiliations:** 1 Research Center for Prevention of Psychosocial Injuries, Ilam University of Medical Sciences, Ilam, Iran; 2 University of Social Welfare and Rehabilitation Sciences, Tehran, Iran.; 3 Social Determinants of Health Research Center, Nursing and Midwifery Faculty, Lorestan University of Medical Sciences, Khorramabad, Iran.; 4 Department of Nursing, Faculty of Nursing and Midwifery, Kurdistan University of Medical Sciences, Sanandaj, Iran.

**Keywords:** Sleep apnea, Meta-analysis, Iran, Prevalence

## Abstract

**Background:**

Sleep apnea is a common sleep disorder which is associated with cardiovascular diseases, diabetes and stroke. Different studies conducted in Iran have reported different prevalence for sleep apnea. The aim of the present study was to determine the prevalence of sleep apnea in Iran.

**Materials and Methods:**

In this study, 42 studies that have been published in Farsi and English languages were selected with no time limit up to the March of 2018. Article search was conducted using “prevalence”, “frequency”, “sleep apnea” and “obstructive sleep apnea” keywords in Scientific Information Database (SID), MagIran, Google Scholar, Science Direct, PubMed and Scopus databases. Data were analyzed using meta-analysis and random effect model methods. Heterogeneity between the studies was evaluated using I^2^ test. Data were analyzed using Stata software version 11.2.

**Results:**

The total prevalence of metabolic syndrome was 44% (95% CI: 35% to 53%). The highest prevalence of sleep apnea distinguished by the disease belonged to patients with sleep disorders (74%, 95% CI: 66%–82%), diabetes mellitus (61%; 95% CI: 46%–76%) and cardiovascular disease (55%; 95% CI: 47%–63%).

**Conclusion:**

Given high prevalence of sleep apnea in Iran, identifying people at risk and providing instructional materials for controlling and treating sleep apnea is necessary.

## INTRODUCTION

Obstructive Sleep Apnea (OSA) is a sleep disorder that is characterized by recurrent episodes of partial or complete obstruction in the upper airways or recurrent arousals during sleep (1). Sleep apnea can lead to several problems, such as morning headaches, fatigue, impairment in daily functioning, memory loss, depression, and impotence in men, and is also related to more serious problems, such as cerebrovascular disease, cardiovascular disease, and motor vehicle accidents (2). Various studies have shown that untreated sleep apnea due to intermittent hypoxia, increased sympathetic nervous system activity, and changes in the chest cavity pressure is related to stroke, cardiac arrhythmia, cardiovascular disease, diabetes mellitus, increased blood pressure, and reduced quality of life (3–8). Because during airway obstruction most patients with sleep apnea are asleep and not aware of their apnea, this condition is often remains undiagnosed (9,10).

Chance of death or cardiovascular disease in patients with sleep apnea is estimated to be 2.5 and 4.5, respectively (11). Old age, smoking cigarette, alcohol consumption, male gender, menopause, race, facial abnormalities, and nasal obstruction are among the other risk factors for sleep apnea (3,12). Sleep apnea can occur at any age; one in every 5 adults has mild sleep apnea and one in every 15 adults has moderate sleep apnea (13). Different prevalence rates have been reported for sleep apnea by different studies and in different samples. In the study by Reddy et al., the prevalence of sleep apnea was 9% among the general population, and twice this percentage among those with obesity (14). The prevalence of sleep apnea is higher in patients than the general population. According to Butt et al study, the prevalence of sleep apnea among patients with Congestive Heart Failure (CHF), renal failure, and stroke is 40, 50, and 60%, respectively (15).

Given that taking any measure to prevent or treat sleep apnea requires accurate estimation of the prevalence of this disorder, we tried to conduct a study on the prevalence of sleep apnea in Iran.

## MATERIALS AND METHODS

This review protocol is registered in the International Prospective Register of Systematic Reviews (PROSPERO) with the number, CRD42017064337. The present study reviews the prevalence of sleep apnea in the Iranian population based on reports from articles published in domestic and international journals, without any time limitation, until March, 2018. International and domestic databases, such as Scientific Information Database (SID), MagIran, Google Scholar, Science Direct, PubMed, and Scopus were searched to find related articles. The following keywords and combinations of them were used to search the databases: Sleep apnea and Sleep breathing disorder. In the Persian databases, Persian equivalents of the keywords were used. In addition, articles’ reference lists were reviewed to find other studies related to the topic.

### Article selection and data extraction

First, we collected all articles in which the keywords had been mentioned. Based on suggestion for reducing publication bias (16), all observation studies with a sample size of above 60 were included in the study. Only the studies were selected that met the inclusion and exclusion criteria. The exclusion criteria were as follows: unrelated to the topic, using treatment interventions for patients with sleep apnea, and lack of access to article full text. Two researchers independently examined article titles and abstracts based on the inclusion and exclusion criteria, separated the related contents, and extracted the full texts of the articles. If the two researchers disagreed on the selection of an article, the final decision was made by the correspondent author who is expert in meta-analysis. The methodological quality of the studies was examined using an instrument commonly used in the Iranian and non-Iranian studies. This instrument assessed 5 aspects of the articles, including study design, comparison group, describing the characteristics of participants, sample size, and detailed description of the instruments used to collect data. A score from 0 to 3 was assigned to each aspect. Articles with a score from 0 to 5 were regarded as having poor methodological quality, 6 to 10 as having average methodological quality, and above 10 as having strong methodological quality (17,18). In order to analyze the articles, a form was used that asked about the following information: Name of the first author, articles’ country of publication, articles’ year of publication, sample size, and number of people with sleep apnea in the population studied. Article selection and screening was based on the PRISMA statement (19). Finally a total of 42 articles were selected for the analysis.

### Statistical analysis

Because prevalence rate has a binomial distribution, the variance for each study was calculated through calculating the variance for binomial distribution. Weighted means were used to aggregate the prevalence rates reported by different studies, and the weight assigned to each study was its inverse variance. The I^2^ index was used to examine the heterogeneity of the data. Heterogeneity was classified into three categories: less than 25% (low heterogeneity), 25 to 75% (moderate heterogeneity) and more than 75% (high heterogeneity). Due to heterogeneity of the data, a random effects model was used to aggregate the studies and for the joint estimation of the prevalence rate. The meta-regression analysis was used to examine the relationship of the prevalence of sleep apnea with article year of publication and sample size. The Begg’s test was used to examine publication bias. All analyses were performed using Stata software, version 11.2.

## RESULTS

**Figure 1.**
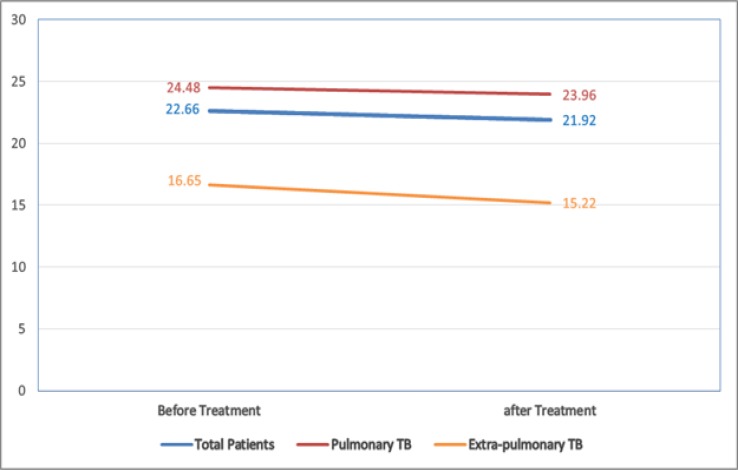
Screening flowchart and selection of qualified articles according to PRISMA guidelines

In this systematic review and meta-analysis, all articles published in Persian and English, aimed at examining the prevalence of sleep apnea, were reviewed based on the PRISMA statement and without any time limitation. In the primary search, a total of 128 articles were identified, among which 86 articles were excluded from the final analysis based on the inclusion and exclusion criteria. The flowchart showing the process of article selection is presented in Figure (1).

**Figure 2.**
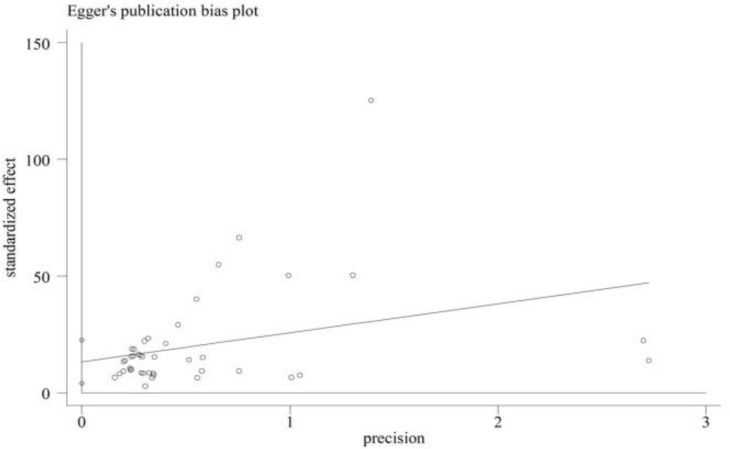
Publication bias in reviewed articles. Due to the fact that most of the points are located outside the 95% confidence interval, the effect of the publication bias is significant.

The articles selected for meta-analysis were examined for sensitivity. The findings indicated that elimination of none of the studies changed the overall estimation of the prevalence of depression. A Bias diagram was used to examine whether all studies on depression among hemodialysis patients in Iran were included in the study. According to the Egger’s regression test, the publication bias was not significant (P=0.006) (Figure 2).

The final articles were from 2011 to March 2018, and the total sample size under was 27684 (mean=660). More than half of the studies (52%) were from 2015 and 2016. In terms of methodological quality, 9 articles were low and the remaining articles were moderate. Other characteristics of the studies are presented in Table 1.

**Table 1.** Characteristics of the selected articles

**No.**	**First Author**	**Year**	**City**	**Sample size**	**Target Group**	**Screening Tool**
1	Khazaie et al. (20)	2018	Kermanshah	140	Depressed patients	BQ
2	Foroughi et al. (21)	2017	Tehran	4021	General population	STOP-BANG
3	Joorabbaf Motlagh et al. (22)	2017	Tehran	643	Drivers	STOP-BANG
4	Ghanei Gheshlagh et al. (23)	2016	Saghez	200	The elderly with cardiovascular disease	STOP
5	Saraei et al. (24)	2016	Tehran	1743	Drivers	STOP-BANG
6	Mohammadi et al. (25)	2016	Yazd	91	Sleep disorders	PSG
7	Farajzadeh et al. (26)	2016	Saghez	175	The elderly	BQ
8	Khajeh-Mehrizi et al. (27)	2016	Tehran	210	Cardiovascular disease	STOP-BANG
9	Khaledi-Paveh et al. (28)	2016	Kermanshah	100	Sleep disorders	PSG
10	Khaledi-Paveh et al. (28)	2016	Kermanshah	100	Sleep disorders	BQ
11	Seyedmehdi et al. (29)	2016	Tehran	715	Hospital staff	BQ
12	Farajzadeh et al. (30)	2016	Saghez	175	Healthy older adults	BQ
13	Farajzadeh et al. (30)	2016	Saghez	175	Depressed older adults	BQ
14	Mozafari et al. (31)	2015	Qom	194	Cardiovascular disease	BQ
15	Naini et al. (32)	2015	Isfahan	200	kidney transplant	BQ
16	Naderan et al. (33)	2015	Tehran	616	Keratoconus	BQ
17	Naderan et al. (33)	2015	Tehran	616	Healthy	BQ
18	Ghajarzadeh et al. (34)	2015	-	82	Pregnant women	BQ
19	Ghazal et al. (35)	2015	Isfahan	127	Cardiovascular	BQ
20	Sadeghniiat-Haghighi et al. (36)	2015	Tehran	122	Diabetes	STOP-BANG
21	Sadeghniiat-Haghighi et al. (37)	2015	Tehran	603	Sleep disorders	STOP
22	Sadeghniiat-Haghighi et al. (37)	2015	Tehran	603	Sleep disorders	STOP-BANG
23	Sadeghniiat-Haghighi et al. (37)	2015	Tehran	603	Sleep disorders	PSG
24	Dehghani et al. (38)	2015	Shahroud	312	Driver	STOP-BANG
25	Khazaie et al. (39)	2015	Kermanshah	448	Healthy	BQ
26	Amra et al. (40)	2014	Isfahan	61	Cardiovascular surgery	BQ
27	Javadi et al. (41)	2014	-	406	Cardiovascular disease	BQ
28	Khazaie et al. (42)	2014	Kermanshah	170	Drivers	-
29	Mozafari et al. (43)	2014	Qom	214	Drivers	BQ
30	Zeighami Mohammadi et al. (44)	2014	Karaj	200	Pregnant women	STOP-BANG
31	Sadeghniiat-Haghighi et al. (45)	2013	Tehran	173	Diabetes	STOP-BANG
32	Ansarin et al. (46)	2013	Tabriz	5545	Healthy	-
33	Nouri-Mahdavi et al. (47)	2013	-	77	Erection problem	PSG
34	Baghi et al. (48)	2013	Saghez	140	Pregnant	BQ
35	Ghanei and Mahmoodi (49)	2013	Saghez	300	Cardiovascular disease	BQ
36	Ghanei Gheshlagh et al. (50)	2013	Saghez	100	Diabetes	BQ
37	Rezaei et al. (51)	2012	Saghez	132	Stroke	BQ
38	Asghari et al. (52)	2012	-	502	Sleep disorders	ESS
39	Khazaie (53)	2011	Kermanshah	527	Healthy	BQ
40	Amra (54)	2011	Isfahan	2462	Diabetes	BQ
41	Amra (55)	2011	Isfahan	3529	Healthy	-
42	Ghanei Geshlagh (56)	2011	Saghez	132	Dialysis	BQ

BQ: Berlin Questionnaire; ESS: Epworth Sleepiness Scale; PSG: Polysomnography

**Figure 3.**
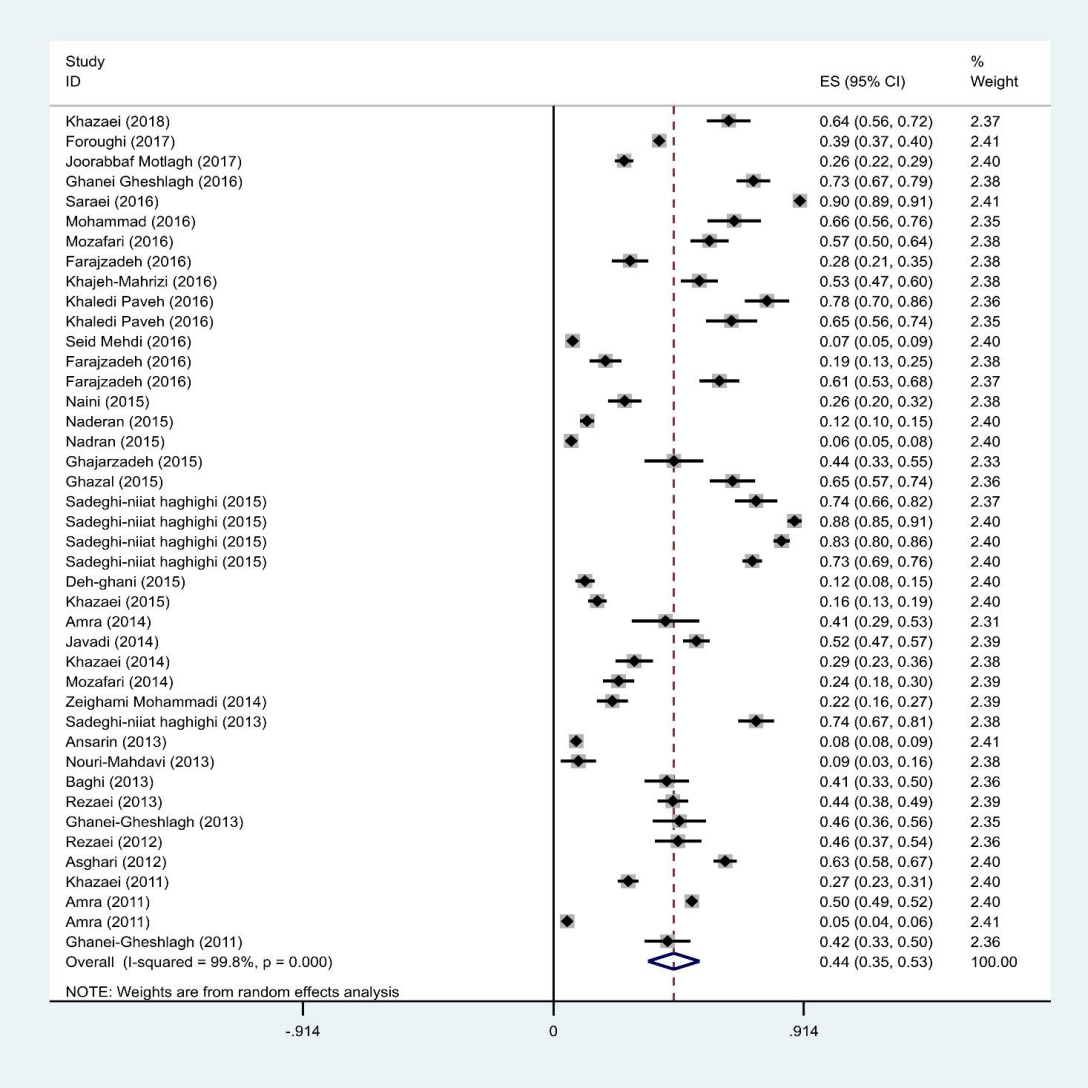
Forest plot of the prevalence of OSA in Iranian people. The confidence interval of 95% for each study in the form of horizontal line around the main mean and the dotted line in the middle represents the mean overall score and the rhombus shows the confidence interval of the prevalence of this disorder.

The prevalence of sleep apnea in Iran was estimated using a random effects model, and was found to be 44% (95% CI: 35–53) in 37 studies with a total sample size of 27684. The heterogeneity in the present study was 99.8% that puts it among studies with high heterogeneity. Therefore, in the next examination, the random effects model was used. The random effects model supposes that observed differences are due to different sampling methods and different prevalence rates reported by different studies. Results based on target population indicated that the highest prevalence of sleep apnea was among people with sleep disorders (74% with 95% CI: 66–82), people with diabetes (61% with 95% CI: 46–76) and people with cardiovascular disease (55% with 95% CI: 47–63) (Figure 3).

Based on Iran’s regions, the highest prevalence of sleep apnea was in the regions 1 and 4 (each 46%), and based on the instruments used to gather data, the highest prevalence rates had been assessed using the Epworth Sleepiness Scale (ESS), the STOP, and the STOP-BANG questionnaire (58%).

The Berlin Questionnaire (BQ) had been used to examine sleep apnea by more than half of the studies. The prevalence rates of sleep apnea based on Iran’s regions, target populations, and instruments used for diagnosis and screening are presented in the following table 2.

**Table 2.** Prevalence of sleep apnea in subcategories of the data

**Groups**		**Number of articles**	**Sample size**	**Prevalence**	**95% confidence interval**	**Heterogeneity**	

						**Percentage**	**P-value**
**Region**	1	16	11588	46	28–64	99.9	0.001
	2 and 5	6	6470	42	17–68	99.8	0.001
	3	10	7074	41	24–58	99.1	0.001
	4	6	1485	46	28–65	98.5	0.001
	Unknown	4	1068	42	19–65	99.8	0.001
**Targer group**	Drivers	5	3082	36	(−4) −77	99.9	0.001
	Cardiovascular disease	7	1498	55	47–63	90.3	0.001
	Sleep disorder	7	3602	74	66–82	95.7	0.001
	Renal disease	2	332	34	18–49	88.6	0.003
	Pregnant women	3	422	35	20–51	91.1	0.001
	Diabetes	4	2757	61	46–76	96	0.001
	Others	14	16991	24	18–31	99.4	0.001
**Scale**	STOP, STOP-BANG, ESS	12	9332	58	40–75	99.8	0.001
	Berlin	23	8237	38	30–47	99	0.001
	PSG	4	871	56	24–89	99	0.001
**Quality**	Low	9	7020	47	24–70	99.5	0.001
	Moderate	33	20664	43	31–55	99.8	0.001

**Region 1:** Alborz, Tehran, Qazvin, Mazandaran, Semnan, Golestan, and Qom.

**Region 2:** Isfahan, Fars, Bushehr, Hormozgan, Kohgiluyeh and Boyer-Ahmad, and Chaharmahal and Bakhtiari

**Region 3:** West Azerbaijan, East Azerbaijan, Ardabil, Zanjan, Gilan, and Kurdistan.

**Region 4:** Kermanshah, Ilam, Lorestan, Hadaman, Markazi, and Khuzestan.

**Region 5:** Razavi Khorasan, North Khorasan, South Khorasan, Kerman, Yazd, and Sistan and Baluchestan

The meta-regression model indicated no significant relationship between sleep apnea and sample size (P=0.128) with methodological quality (P=0.117). The meta-regression results showed an increase in the prevalence of sleep apnea from 2010 to 2018, however this trend was not significant (P=0.158). Examination of publication bias using the Egger’s regression test indicated that the publication bias of the studies was not statistically significant (P=0.006).

## DISCUSSION

In this systemic review and meta-analysis, a total of 42 studies conducted from 2011 to 2018 were reviewed. According to the results, the prevalence of sleep apnea in Iran was 44% (95% confidence interval: 35–53). The prevalence of sleep apnea has been reported to be 33% in Saudi Arabia (57) and 12.4% in Pakistan (58), therefore, the prevalence of this condition is lower in these countries than in Iran. One factor that may explain the high prevalence of sleep apnea in Iran is the high prevalence of obesity in the Iranian population. The chance of having sleep apnea becomes twice with 10 kilograms increase in weight, and 4 times with 15 centimeters increase in the waist size (26).

In a review study, Mirrakhimov et al. examined the prevalence of sleep apnea in Asia, and found that the highest prevalence of sleep apnea was in the Iranian population (27.3%) (59). In the meta-analysis conducted in Asia (in contrast with the present review), only studies published in English with a sample size of above 100 (in case of using polysomnography) and above 300 (in case of using screening questionnaires) were analyzed. In addition, the search for articles had only been done in international databases. Although obesity is less prevalent among Asians than in the American and European countries, the prevalence of sleep apnea is higher in Asia than in western countries, indicating a relationship between race and sleep apnea (60). Lam et al. believe that the higher prevalence of sleep apnea among the Asian race is due to their craniofacial features (61).

The study results based on the type of illness showed that the highest prevalent of sleep apnea was among people with sleep disorders, people with diabetes (61% with 95% CI: 46–76), and people with cardiovascular disease (55% with 95% CI: 47–63), respectively. Foster et al. showed that 86% of patients with diabetes also had sleep apnea (62). The high prevalence of sleep apnea among those with diabetes can be explained by obesity and plaque buildup in their upper airways. The prevalence of sleep apnea among patients with heart attack and cardiomyopathy has been reported to be 70% (63,64) and 35% among those with congestive heart failure (65). Recurrent congestions of the airways with imposing negative pressure inside thorax, hypoxia hypercapnia, and vasoconstriction lead to increase in afterload and cardiovascular disease (49).

The highest prevalence of sleep apnea had been assessed using the following instruments, respectively: The Epworth Sleepiness Scale (ESS), the STOP-BANG, and the STOP (58% with 95% CI: 40–75) and the lowest prevalence had been assessed using the BQ (37% with 95% CI: 30–47). Polysomnography is the gold standard in diagnosis of sleep apnea. The analyses using polysomnography indicated that the prevalence of sleep apnea in Iran was 56% (with 95% CI: 24–89). The ESS has eight items assessing daytime sleepiness in different conditions, including during reading, watching television, sitting in public places, being in a car as a passenger, immediately after lunch, lying down after lunch, talking with others, and waiting behind the red-light. One of limitations of the ESS is that some of its items are not suitable for people with hearing or vision problems (for example, illiterate people cannot read the first item of the scale). The STOP assesses four conditions, including snoring, daytime tiredness, others hearing one’s snoring, and hypertension, with Yes/No questions. Giving Yes to two to more items indicates sleep apnea. In addition to the four previously-mentioned items, the STOP-BANG, assesses four demographic criteria, including Body Mass Index (BMI) over 35 kg/m^2^, age over 50 years old, neck circumference over 43 centimeters in men and over 41 centimeters in women, and being male. Having more than four of the criteria indicates sleep apnea (66). These two instruments are more objective than the ESS. The BQ has ten items and three dimensions (snoring, daytime sleepiness, and hypertension/BMI). If a respondent gives a Yes to two or more dimensions, he/she is considered to be at high risk for developing sleep apnea (30). The prevalence of sleep apnea in the studies that had used polysomnography was 56% (with 95% CI: 24–89). Polysomnography is the gold standard in diagnosis of sleep apnea, but it is time-consuming, not easy to use, not easily accessible, and expensive; therefore, other instruments are often used to diagnose sleep apnea which allow for identification of those at high risk for developing this condition in different setting, such as home or hospital (5,66).

Due to its high prevalence, sleep apnea has become an important and critical challenge for the general health of the Iranian population. Early diagnosis of those at high risk for developing this condition, and providing educational materials aimed at controlling and treating sleep apnea seem to be important and necessary.
